# Distal Clavicle Osteolysis after Modified Weaver-Dunn's Procedure for Chronic Acromioclavicular Dislocation: A Case Report and Review of Complications

**DOI:** 10.1155/2014/953578

**Published:** 2014-12-02

**Authors:** Eduard Alentorn-Geli, Fernando Santana, Felipe Mingo, Ignasi Piñol, Albert Solano, Lluís Puig-Verdié, Carles Torrens

**Affiliations:** ^1^Department of Orthopedic Surgery and Traumatology, Parc de Salut Mar, Hospital del Mar and Hospital de l'Esperança, Universitat Autònoma de Barcelona (UAB), Passeig Maritim 25-27, 08003 Barcelona, Spain; ^2^Department of Radiology, Parc de Salut Mar, Hospital del Mar and Hospital de l'Esperança, Universitat Autònoma de Barcelona (UAB), Passeig Maritim 25-27, 08003 Barcelona, Spain

## Abstract

Distal clavicle osteolysis after acromioclavicular joint stabilization has only been described after the use of hardware for clavicle stabilization or synthetic graft causing a foreign body reaction. This paper reports a very rare case of distal clavicle osteolysis after modified Weaver-Dunn procedure for the treatment of chronic acromioclavicular joint dislocation. The paper also provides a comprehensive review of complications of this surgical technique and discusses a potential vascular etiology and preventive strategies aimed at avoiding clavicle osteolysis.

## 1. Introduction

While surgical treatment of acute acromioclavicular (AC) dislocation remains controversial, painful symptomatic chronic injuries usually require surgical management. Modified Weaver-Dunn's technique is a well-known surgical procedure for type III AC joint dislocation [1]. This technique provides adequate stabilization of AC joint with satisfactory clinical outcomes and low complication rate [2–4]. Most common complications include infection, clavicular erosion, neurovascular injury, continued pain, coracoclavicular calcification, keloids, cosmetic complaints, coracoid fracture, aseptic foreign-body reaction, AC osteoarthritis, or AC joint instability [4–8].

Well-known causes of clavicle osteolysis are acute trauma or chronic overuse [9, 10]. Specifically, it has been reported that direct traumatism over the AC joint [10] or overuse due to weight lifting [9, 10] can cause distal clavicle osteolysis. To the best of our knowledge, the development of clavicle osteolysis after AC joint stabilization has only been reported related to the use of hardware or Gore-Tex (W. L. Gore Associates, Flagstaff, AZ) graft fixation [11, 12] but not the use of the modified Weaver-Dunn's technique. The purpose of this paper is to report a rare case of clavicle osteolysis after AC joint stabilization using the modified Weaver-Dunn's technique in a chronic painful AC joint dislocation.

## 2. Case Presentation

A 49-year-old man, with unremarkable past medical history, came to our clinic in 2009 after falling onto his right shoulder. The patient's chief complaints consisted of right shoulder pain upon movements and deformity. Physical examination revealed tenderness and deformity at the AC joint, exacerbated with arm abduction in the coronal plane and forced adduction in the transverse plane. Plain radiographs showed a superior displacement of the clavicle of more than 50% compared to the normal side, corresponding to a type III AC joint dislocation according to Rockwood's classification [13]. He was recommended to be initially treated conservatively with a sling in internal rotation for three weeks. Then, the patients followed a rehabilitation program to gain shoulder range of motion and strength. After 6 months, the patient was scheduled for surgery because of persistent pain despite rehabilitation.

A modified Weaver-Dunn's technique was planned to address this chronic type III AC joint dislocation. Standard patient positioning and surgical approach were employed. Distal clavicle excision and transposition of the coracoacromial ligament to the clavicle were performed. An absorbable anchor using the Panalok system (DePuy Mitek, Inc., Raynham, Massachusetts, USA) was placed at the base of the coracoid process and sutures were passed through drill holes in the clavicle to ensure primary stabilization of the AC joint.

**Figure 1:**
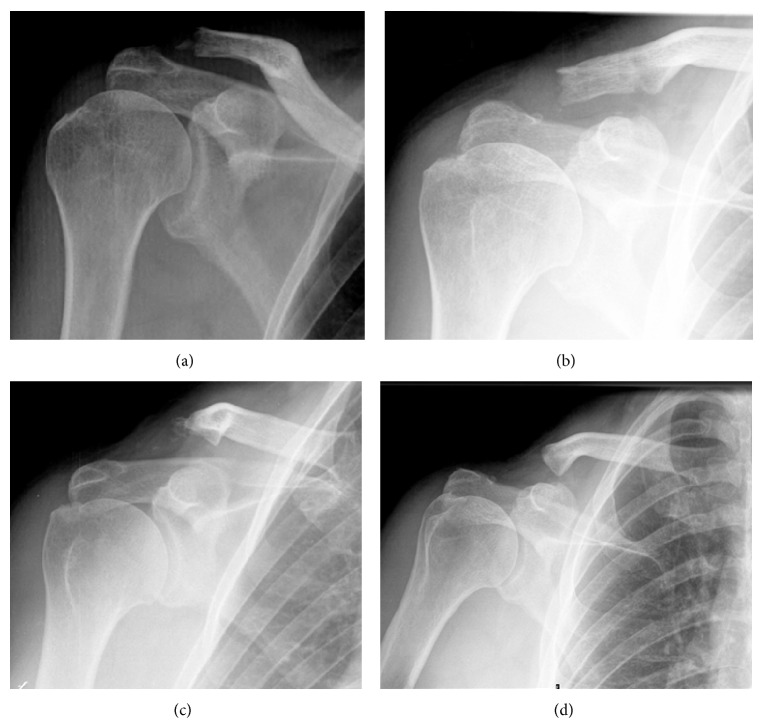
Serial of plain radiographs during the follow-up demonstrating the progression of the distal clavicle osteolysis. (a) 2 weeks after surgery. (b) 2 months after surgery. (c) 1 year after surgery. (d) 2 years after surgery.

**Figure 2:**
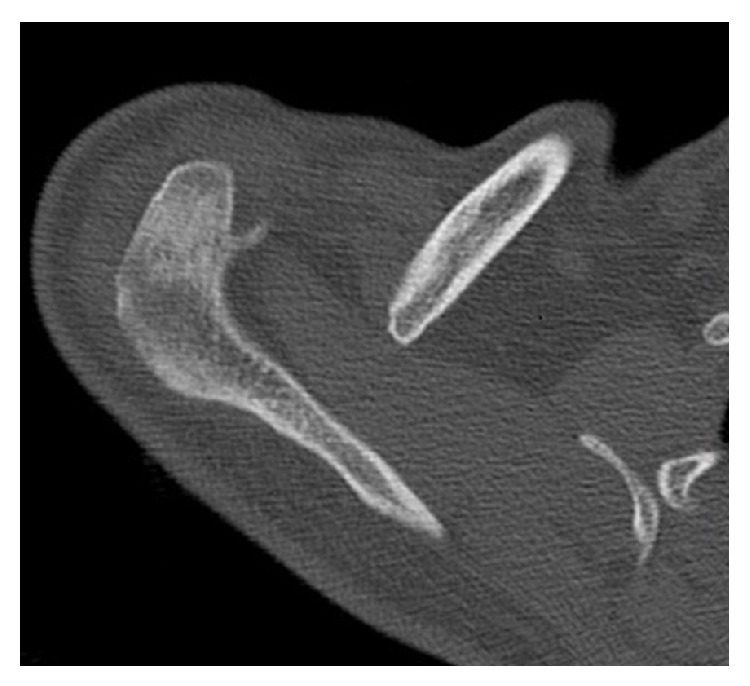
CT scan of the acromioclavicular joint demonstrating distal clavicle osteolysis compared to a normal joint.

**Figure 3:**
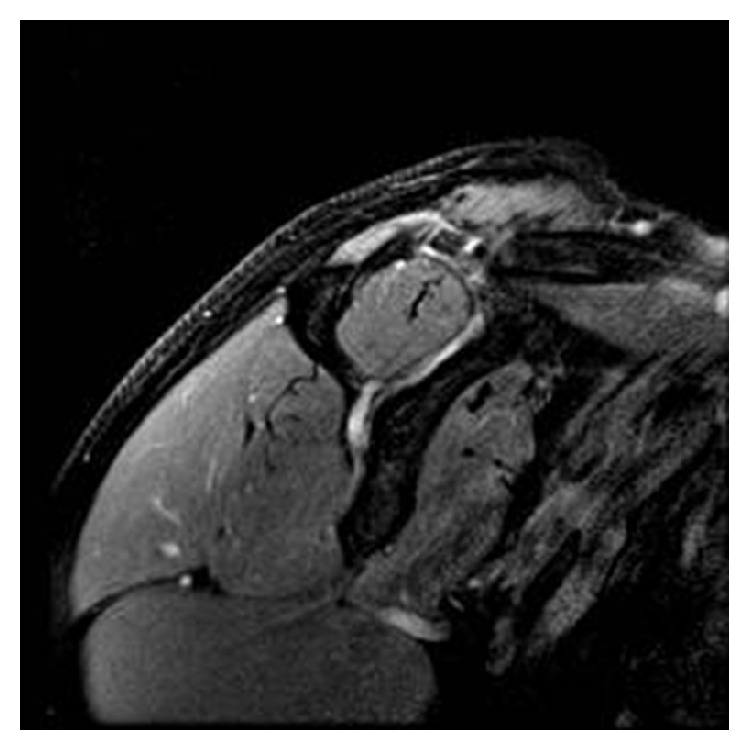
Magnetic resonance imaging of the acromioclavicular joint in DP fat-suppression sequence demonstrates the osteolysis with no soft tissue reaction or bone edema.

Plain radiographs 2 weeks and 2 months after surgery demonstrated correct reduction of the AC joint, adequate bone appearance of the clavicle, and no signs of hematoma and infection. In the follow-up visit 6 months after surgery, the patient complained of AC joint pain on palpation but demonstrated full active range of motion. One year after surgery, shoulder function was also complete, but pain was still present on palpation. Plain radiograph at that time showed initial osteolysis of the clavicle. Two years after surgery, the patient's shoulder symptoms and function did not change, but radiographs demonstrated progression of osteolysis (Figure 1). A CT scan confirmed the lateral third clavicle osteolysis and found no associated bone injuries (Figure 2). MRI study confirmed the osteolysis with no bone edema or soft tissue reaction (Figure 3). Despite image findings, the patient currently refers no clinical symptoms except a minimum loss of strength in specific movements over the head. However, the patient is pain free and declines further surgery.

## 3. Discussion

We report an unusual case of clavicle osteolysis following stabilization of the AC joint because of dislocation. This patient underwent a modified Weaver-Dunn's procedure where a restoration of the function of coracoclavicular ligaments was established with the transposition of the coracoacromial ligament plus the use of an absorbable anchor. This technique has demonstrated good clinical outcomes and low complication rate [2–4]. In fact, the modified Weaver-Dunn's technique is a common procedure for the treatment of AC joint dislocation [1, 3, 14–22].

Distal clavicle osteolysis is a rare disorder that may be caused by acute trauma, chronic overuse, systemic disorders (hyperparathyroidism, connective tissue disorders, and infection), or surgery [9–12, 23–25]. One of the most accepted theories explaining distal clavicle osteolysis secondary to chronic overuse is the development of stress fractures on the subchondral bone from repetitive microtrauma. This would create subchondral fissuring, osteolysis, and an attempted repair through an increased osteoblastic activity [23]. This might occur along with degenerative joint disease of the AC joint, where cartilage and subchondral damage precedes the migration of synovium and synovial fluid to the subchondral bone. Postoperative osteolysis has been mainly related to the use of hardware. Eskola et al. observed this phenomenon in 15% of patients (13 out of 86) undergoing acromioclavicular ligament suture plus Kirschner wire or cortical screw fixation for complete AC joint dislocation [11]. The development of clavicle osteolysis without the use of hardware has also been reported in a patient with AC joint reconstruction using a Gore-Tex (W. L. Gore Associates, Flagstaff, AZ) graft [12]. In this case, the osteolysis was related to foreign-body reaction [12]. However, there are no reports on clavicle osteolysis as a complication of modified Weaver-Dunn's procedure when excluding studies employing hardware for AC joint stabilization or related to foreign-body reaction [1, 3, 14–22]. Most common complications include (Table 1) minor displacement of the clavicle (range 9.5% to 25%) [1, 14, 17, 18, 22], clavicle redislocation (range 4.3% to 6.6%) [1, 14, 17, 22], AC joint periarticular calcifications (range 10% to 61%) [14, 15, 17, 22], superficial wound infection (range 4.7% to 10%) [17, 18, 20–22], deep infection (range 0% to 6.8%) [18, 19], painful shoulder stiffness (13%) [3], partial bone block union to clavicle (20%) [21], and scar sensitive (6.6%) [3].

The knowledge of the blood supply of the clavicle may help understand the occurrence of nonunion and osteolysis. Knudsen et al. found that the suprascapular, the thoracoacromial, and the internal thoracic arteries provided blood to the clavicle [26]. The authors reported that the main blood supply was primarily periosteal and that no nutrient artery was found. Therefore, any injury to the periosteal vascularity during the surgical procedure may cause clavicle osteolysis or nonunion [5, 26]. Considering that important branches reach the posteroinferior part of the clavicle, care must be taken to not perform a wide deperiostization of the lower rim of clavicle. In fact, the thoracoacromial artery was found to constantly supply the lateral 4/5 of the clavicle through its clavicular and acromial branches, with an inferoanterior relation to the bone approximately 2 cm medial to the distal end [26]. One step of the modified Weaver-Dunn's technique is the oblique resection of 2 cm of the distal clavicle. This step may explain the development of osteolysis or nonunion.

Although most common vascular injuries following AC joint stabilization may involve the great subclavian vessels [5, 6, 8], disruption of small vessels may also occur after modified Weaver-Dunn's procedure leading to osteolysis of the clavicle. Special care must be taken when performing modified Weaver-Dunn's technique not to elevate too much of periosteum from the lateral clavicle, since its main blood supply is provided by the periosteum, and also to avoid damage of clavicular and acromial branches of the thoracoacromial artery.

## Figures and Tables

**Table 1:** Summary of studies reporting complications with Weaver-Dunn's procedure for acromioclavicular dislocation.

Authors	Type of study	Study characteristics	Complications^*^
Weaver and Dunn, 1972 [1]	Case series—Level IV evidence	15 pts; 12 acute, 3 chronic; mean age 31 yo; 12 men, 3 women; 13 acute; 2 chronic; mean F-U 35 mo (range 16–52 mo)	Mild clavicle elevation 20% Redislocation 6.6% Shoulder weakness 0%

Rauschning et al., 1980 [14]	Case series—Level IV evidence	17 pts; 16 men, 1 women; mean age 30 yo (range 15–60 y); 12 acute, 5 subacute or chronic; mean F-U 3 y	Calcifications in ruptured ligaments 50% Mild subluxation <5 mm: 25% (stress radiograph) Redislocation 5.8% (epileptic attack)

Mulier et al., 1993 [15]	Case series—Level IV evidence	58 pts undergoing conservative treatment: 10 failed cases treated with WD; mean age 31 yo (range 17–50 y); F-U 6.4 y	Ossification 10% in operated pts

Bradley and Tibone, 1997 [16]	Case series—Level IV evidence	18 pts; types III, IV, and V ACJ dislocations; mean age 35 yo (range 19–62 y); 11 chronic, 7 acute; mean F-U 39 mo (range 18–77 mo)	No complications reported

Tienen et al., 2003 [17]	Case series—Level IV evidence	21 pts; acute injuries; all type V ACJ dislocations; all competitive athletes; mean age 32 yo; F-U 35 mo (range 4 to 55 mo)	Minor periarticular calcifications in ACJ 28.5% Subluxation of clavicle 9.5% Fully dislocated clavicle 4.7% Superficial wound infection 4.7% Major ossification 0%

Kumar et al., 2007 [3]	Case series—Level IV evidence	15 pts; chronic injuries; mean age 42 yo (range 25–59 y); 13 men, 2 women; 12 heavy physical activity; F-U 26 mo (range 12–64 mo)	Clavicle prominence 13.3% Painful stiffness 13.3% (limitations to IR and ABD) Scar sensitive 6.6% Perioperative complications 0%

Somers and van der Linden, 2007 [18]	Case series—Level IV evidence	12 pts: 10 treated with WD, 2 with simple fixation to coracoid; 4 chronic, 8 acute injury; F-U 6–18 mo; sample characteristics not reported	Minor nonsymptomatic displacement of clavicle (<3 mm) 25% Infections: superficial (wound) 8.3%, deep 0% Loss of fixation 0% Migration of metallic anchors 0%

Bezer et al., 2009 [19]	Case series—Level IV evidence	33 pts with chronic injury; 4 lost; mean age 29.8 yo (range 19–47 y); 21 men, 8 women; all type III ACJ dislocations; F-U 69.4 mo (range 25–143 mo)	Deep infection 6.8% Clavicle displacement 0% NV complications 0%

Tauber et al., 2009 [20]	Cohort study—Level II evidence	24 pts: 12 treated with modified WD, 12 with ST graft; chronic ACJ dislocations; 14 men, 10 women; mean age 42 yo; F-U 37 mo (range 24–58 mo)	WD group: (i) superficial wound infection 8.3%

Boileau et al., 2010 [21]	Case series—Level IV evidence	10 pts; 8 men, 2 women; chronic grades III/IV ACJ dislocations; 3 pts had initial pining in acute phase and 2 Mumford procedures; mean age 41 yo (range 19–52 y); mean F-U 12.9 mo (range 6–20 mo)	Partial bone block union to clavicle 20% Superficial wound infection 10% Endobutton migration 10% Intraoperative complications 0% ACJ instability or recurrence 0% Periarticular calcifications 0%

Boström Windhamre et al., 2010 [22]	Retrospective comparative study—Level III evidence	47 pts: 23 operated with WD + PDS suture, 24 with WD + hook plate fixation; chronic injuries type III-IV-V. WD + Suture: 13 men, 10 women; mean age 39 yo (range 23–56 y); mean F-U 99 mo (range 51–155 mo)	WD + PDS: (i) calcification CA: mild 43.7%, moderate 13%, severe 4.3% (ii) subluxation: <25%: 5 pts (21.7%); 25–100%: 10 pts (43.7%); >100%: 3 pts (13%) (iii) superficial wound infection 8.7% (iv) redislocation: 4.3%

pts: patients; WD: Weaver-Dunn; F-U: follow-up; yo: years old; y: years; ACJ: acromioclavicular joint; NV: neurovascular; ST: semitendinosus; ACL: acromioclavicular ligament; IR: internal rotation; ABD: abduction; CA: coracoacromial ligament.

^*^All reported complications are summarized. Any complication not included means not reported by the authors.
